# Survey-based exploration of menopause transition experiences of female staff employed in UK ambulance services (CESSATION Phase 2)

**DOI:** 10.29045/14784726.2025.3.9.4.7

**Published:** 2025-03-01

**Authors:** Larissa Prothero, Shona Brown, Tessa Noakes, Allan Clark, Theresa Foster

**Affiliations:** East of England Ambulance Service NHS Trust ORCID iD: http://orcid.org/0000-0002-5440-8429; East of England Ambulance Service NHS Trust ORCID iD: https://orcid.org/0009-0003-6510-2622; East of England Ambulance Service NHS Trust ORCID iD: https://orcid.org/0000-0001-5245-3835; University of East Anglia ORCID iD: https://orcid.org/0000-0003-2965-8941; East of England Ambulance Service NHS Trust ORCID iD: https://orcid.org/0000-0002-6395-0885

**Keywords:** ambulances, female, menopause, working conditions, workplace

## Abstract

**Introduction:**

Often women will experience the menopause and its associated symptoms during their working lives, and there is now an increased focus on improving menopause support offerings in the workplace. The main aim of this study was to explore the menopause transition experiences of ambulance staff and identify workplace interventions that may offer improved support to menopausal staff within the ambulance sector.

**Methods:**

A purpose-designed, online survey was disseminated to UK ambulance services for voluntary completion between December 2021 and February 2022. Topics that were covered included participant age and role, menopause transition phase, symptoms experienced and their severity, expectation and effect of symptoms, work pattern, need for work leave and policy awareness. An opportunity for additional menopause-related comments was included. Quantitative data analysis was performed using descriptive and exploratory statistics; qualitative data were analysed using content analysis.

**Results:**

A convenience sample of 1896 survey responses was obtained; the majority of participants self-reported to be in the menopause transition. Pre- and peri-menopausal participants were more likely to undertake shift-based work; menopausal and post-menopausal participants were more prevalent in office-based roles. Shift-working participants reported more severe tiredness or low energy levels, hot flushes, night sweats and gastric problems; office-working staff reported more severe issues with vaginal health, weight, skin changes and oral health. There were significant associations between role type, severity of symptom impact and need for leave from work. Recommended areas for organisational improvement to support menopausal women in the ambulance workplace are alternative work opportunities, menopause education and training, appropriate menopause policies and guidance, expert resources and support and access to improved physical work environments.

**Conclusion:**

Women can experience menopausal symptoms that impact their working lives; their work role and setting can influence their menopause experiences. Workplace interventions for improved menopause awareness, support and staff well-being are warranted.

## Introduction

There is increasing focus on improving the lives of women experiencing the menopause transition across UK workplaces (Department of Health, 2024; Department of Health and Social Care, 2022; Scottish Government 2021; Welsh Government, 2022). The menopause typically occurs between the ages of 45 and 54 years, and menopausal symptoms can have significant personal and work-life impacts for an individual. A need for appropriate menopause support has been recognised because work absence, reduced working hours, not seeking promotion and alternative career changes have been associated with menopausal symptoms (Bazeley et al., 2022; Chartered Institute of Personnel and Development, 2023).

To date there is limited literature about the menopause transition and emergency service personnel. Recently, a large survey of menopausal police officers in England and Wales has highlighted the challenges that officers face in the workplace, particularly with regard to menopause symptom management, job performance, managerial support and education, and lack of menopause policies (Police Federation of England and Wales, 2024). In 2019 Watkins et al. reported menstrual cycle and menopause-related challenges faced by women firefighters; in particular, thermoregulation, fatigue and personal hygiene (Watkins et al., 2019). A recent pilot evaluation, the MIDWEST study, reported the experiences and impacts of the menopause in female ambulance staff employed in a single UK ambulance service. Menopausal symptoms were found to be wide-ranging, with varying levels of impact, which could be significant, affecting both work and home lives (Prothero et al., 2021).

Women now represent almost one half (42.5%) of the UK ambulance workforce, with individuals being employed across all service roles (NHS England, 2021). More than 16,000 female paramedics are currently registered with the Health and Care Professions Council (HCPC) (2023). In addition, many of these women will be aged 40 years or more (HCPC, 2019; NHS Employers, 2019), so likely to begin the menopause transition soon in their working lives. The MIDWEST study findings have been used to inform the UK-wide three-phase research study, CESSATION. Current national ambulance service menopause guidance, policies and support have been reviewed using content analysis and workforce data has been analysed using descriptive approaches (CESSATION Phase 1). Survey and interview activities (CESSATION Phase 2 and 3, respectively) have assisted the understanding of work and personal impacts of the menopause on female ambulance staff and helped with identifying service developments and interventions that may best support menopausal female staff. Phases 1 to 3 occurred concurrently, and the findings of Phase 3 have recently been published (Brown et al., 2023).

The main aim of this article is to report the findings of CESSATION Phase 2, which was a national online anonymous menopause survey, within the context of national ambulance workforce data (Phase 1; see Supplementary 1a). Our objective is to understand the impacts of the menopause for shift- and office-working female ambulance staff.

## Methods

### Phase 2 survey

A purpose-designed, open, online 22-question survey (Supplementary 2) was devised, based on the findings of the East of England Ambulance Service NHS Trust (EEAST) service evaluation, MIDWEST (Prothero et al., 2021), to explore the menopause experiences of shift- and office-working ambulance staff across the UK. The questions covered topics such as length of work for the ambulance service, function of work, working patterns, driving requirements, participant age, stage of menopause, symptoms experienced and their severity, expectation and effect of symptoms, need for time off work and service menopause policy awareness.

#### Researcher characteristics

The research team, including patient and public representatives, comprised individuals who were pre-menopausal, peri-menopausal and post-menopausal, or had a partner in the menopause transition.

#### Context and sampling

The survey was advertised to 12 UK ambulance service staff (EEAST staff were excluded), with study materials (including an access weblink and QR code) being shared with research and development departments for local dissemination using service advertising methods. The survey was advertised to allow a maximum period of 60 days for completion between 1 December 2021 and 28 February 2022. Survey involvement was voluntary and anonymous, and informed consent was received from participants upon completion and submission of the survey. A free-text question was incorporated for additional participant comments or menopause-related service improvement suggestions.

#### Data collection and analysis

All survey responses were securely stored electronically (password protected) via the EEAST Microsoft Forms platform, which automatically collates all information provided as a Microsoft Excel Worksheet. No personally identifiable information was collected, except for an expression of interest to participate in a Phase 3 study research interview. Data analysis was performed using Microsoft Excel and RStudio; quantitative data were analysed using descriptive and exploratory statistical techniques (in particular, the Chi-square test of independence and the Fisher test). Using a positivist approach (participant survey comments being considered empirical data), qualitative content analysis was used to analyse free-text data, grouping related comments into categories (Drisko and Maschi, 2015). Authors LP, TN and SB independently reviewed all comments, generated initial codes and, via an iterative process, identified topic categories based on the coding. Aided by the study patient and public representatives, the authors then reviewed and refined the categories to agree meaningful topic categories.

#### Manuscript

SRQR guidelines and the CHERRIES checklist provided the framework for the preparation of this manuscript (Eysenbach, 2004; O’Brien et al., 2014).

## Results

### Synthesis and interpretation

#### Participant profile

A total of 1914 surveys were received (completion rate: 100%), which, following the removal of confirmed duplicate entries by individual participants (n = 18), provided a convenience sample of 1896 surveys. Ambulance service contributions varied from 2.1% to 15.5% of total sample responses.

All key service areas were represented by participants: emergency operational delivery (57%; n = 1085), support roles (17%; n = 325), ambulance operations centre roles (16%; n = 309), scheduled patient transport (6%; n = 110) and resilience and specialist operations (1%; n = 25). A minority of participants preferred not to state their service role (2%; n = 42). In agreement with this profile, more than three-quarters of participants (78%; n = 1472) reported working shifts or unsocial hours, with the remainder (22%; n = 424) working regular office hours.

Approximately one third of participants (shift-working: 35%, n = 516; office-working: 32%, n = 134) self-reported to be in the peri-menopause, 30% (shift-working: n = 435; office-working: n = 126) had reached the menopause and approximately 13% (shift-working: n = 169; office-working: n = 65) were post-menopausal. The remainder of participants were not sure which transition phase they were in (shift-working: 10%, n = 151; office-working: 10%, n = 43) or reported to be pre-menopausal, that is, had not yet started this life phase (shift-working: 13%, n = 201; office-working: 13%, n = 56).

**Figure 1:**
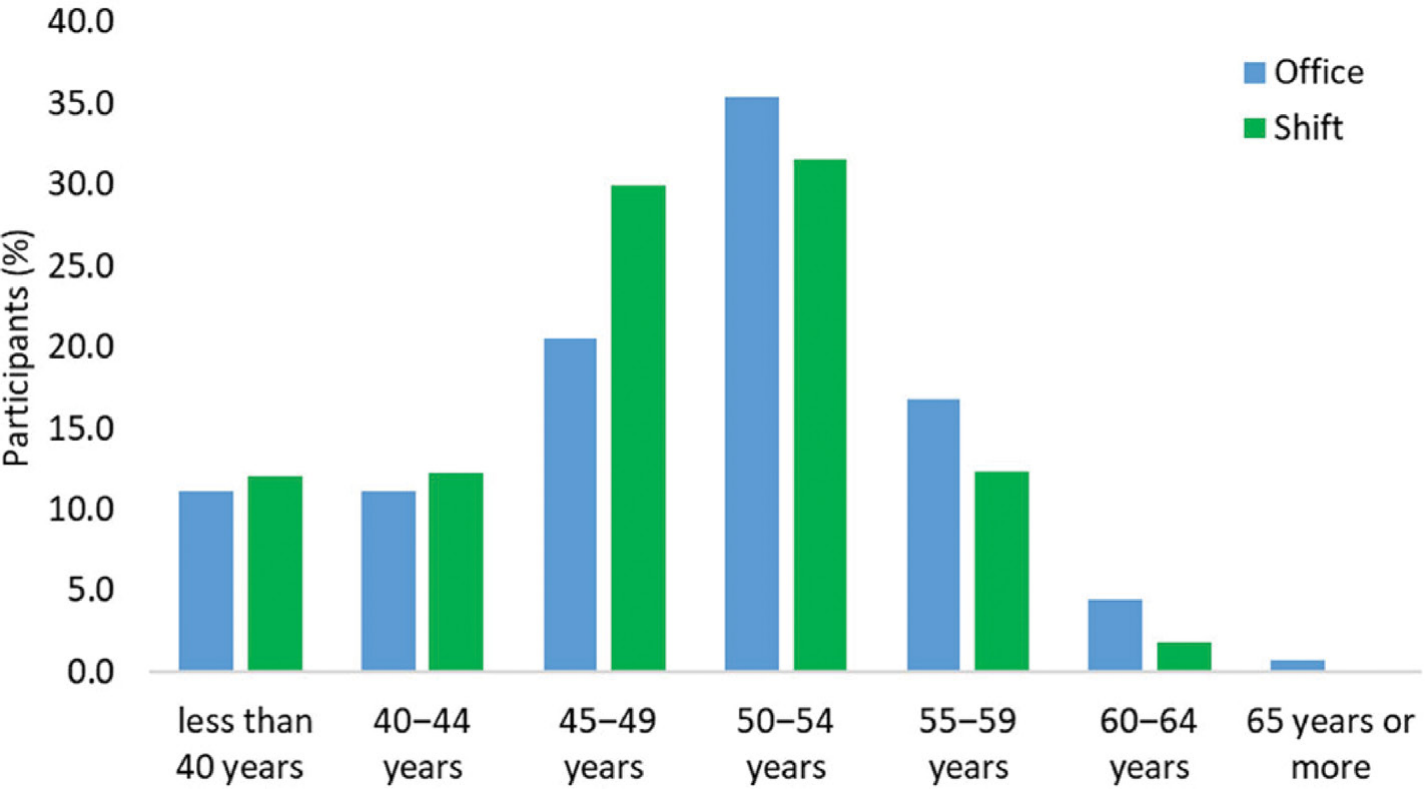
Figure 1. Age profile of CESSATION survey participants by role type.

Regardless of work pattern, the participant ages ranged from less than 40 years to more than 65 years. Pre- and peri-menopausal individuals (those typically less than 50 years) were more likely to undertake shift-working, whereas menopausal and post-menopausal participants (those typically 50 years and older) appeared more prevalent in office-based roles (see Figure 1).

#### Menopause transition

**Figure 2:**
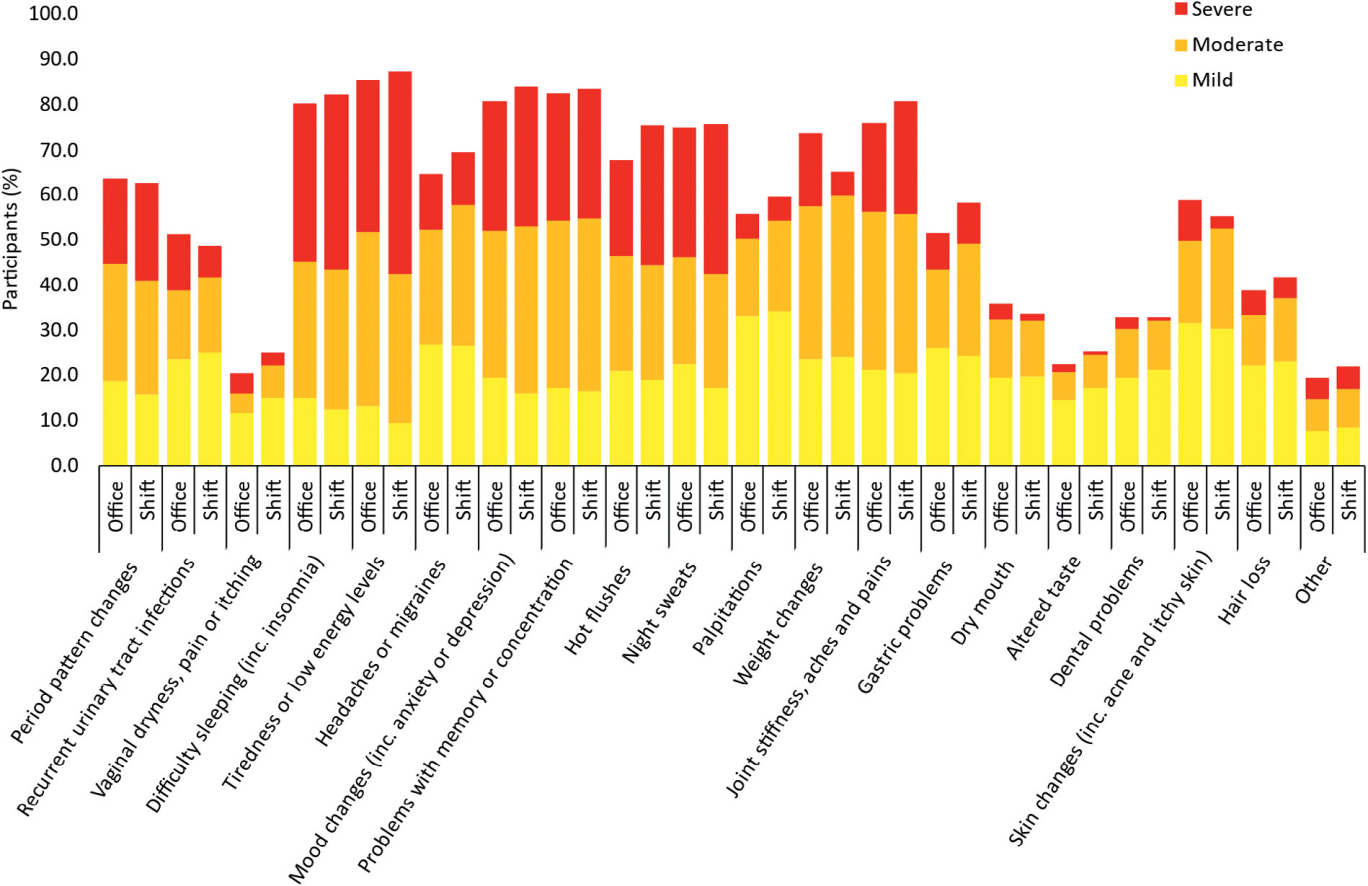
Figure 2. Menopausal symptoms and their severity as experienced by office- and shift-working CESSATION participants.

The majority of participants in the menopause transition experienced numerous and varied menopausal symptoms (86%; n = 1626; Figure 2). Statistical analysis revealed a significant association between role type (office-working and shift-working) and increased severity of menopause symptoms (see Table 1).

**Table 1.** Association between role type (office-working and shift-working) and increased severity of menopause symptoms. When symptoms are experienced, their severity (mild, moderate or severe) is significantly associated with role type: *p <0.05.

Menopause symptom	df	χ^2^	n	p
Tiredness or low energy levels	2	18.257	1650	<0.001*
Difficulty sleeping, including insomnia	2	3.253	1551	0.197
Mood changes, including anxiety or depression	2	4.316	1579	0.116
Problems with memory or concentration	2	0.205	1577	0.903
Joint stiffness, aches or pains	2	3.035	1512	0.219
Night sweats	2	7.157	1430	0.028*
Hot flushes	2	8.902	1396	0.012*
Period pattern changes	2	2.992	1192	0.224
Weight changes	2	45.884	1269	<0.001*
Headaches or migraines	2	2.125	1297	0.346
Gastric problems	2	6.358	1076	0.042*
Skin problems, including acne and itchy skin	2	35.95	1062	<0.001*
Palpitations	2	1.131	1113	0.568
Vaginal dryness, pain or itching	2	14.132	933	<0.001*
Hair loss	2	1.548	778	0.461
Dry mouth	2	8.540	646	0.014*
Dental problems	2	8.156	625	0.017*
Recurrent urinary tract infections	2	4.851	456	0.883
Altered taste	2	5.791	467	0.553
Other	2	0.143	404	0.931

Of the participants experiencing menopausal symptoms, approximately 40% reported not knowing what to expect (shift-working: 40%, n = 593; office-working: 39%, n = 166) and one third reported that their symptoms were worse than they had expected (shift-working: 35%, n = 508; office-working: 31%, n = 132). Approximately one in 10 stated that their symptoms were better than they had expected (shift-working: 8%, n = 118; office-working: 12%, n = 51).

#### Menopause impact

There was a significant association between role type and the severity of symptom impact on well-being (χ^2^(4, n = 1582) = 25.97, p <0.001) and work-life (χ^2^(4, n = 1582) = 38.93, p <0.001). The majority of shift- and office-working staff agreed or strongly agreed their symptoms impacted on their life. There was a significant association between role type and the need for time off work: shift-working staff were more likely to require leave compared with office-working colleagues (χ^2^(1, n = 1582)= 12.03, p = 0.001). Furthermore, only approximately half of the individuals requiring leave told their manager the real reason for their work absence (shift-working: 48%, n = 179; office-working: 54%, n = 40).

#### Professional menopause support

Approximately two-thirds of menopause transition participants (shift-working: 68%, n = 869; office-working: 63%, n = 231) reported they had sought professional healthcare advice. Of this subgroup, the majority had sought general practitioner (GP) support (shift-working: 98%, n = 848; office-working: 95%, n = 219), almost one in five individuals had sought obstetric-gynaecological support (shift-working: 16%, n = 142; office-working: 20%, n = 46) and one in 10 had accessed specialist online guidance (shift-working: 10%, n = 87; office-working: 11%, n = 26). A further 6% had seen a British Menopause Society-recognised menopause specialist (shift-working: 5%, n = 44; office-working: 7%, n = 16).

#### Symptom management

Shift- and office-working participants (n = 1109) shared their personal symptom management strategies, the main categories and subcategories being summarised in Table 2. Five main categories were identified: prescribed medications, over-the-counter treatments, invasive interventions, lifestyle changes and temperature management.

**Table 2.** Categories and subcategories of symptom management strategies used by menopausal CESSATION participants.

Categories	Subcategories
Prescribed medications	Anti-depressant and anxiety medication
	Hormone replacement therapy / hormone medication
	Skin treatments / topical creams
	Antifibrinolytic/menorrhagia treatments
	Gastric/urinary medications
	Headache/migraine medications
	Sleep medications
Over-the-counter treatments	Menopause support
	Herbal supplements
	Skin treatments/creams
	Analgesics/anti-inflammatories
	Probiotics
	Vitamin/mineral supplements
	Sleep support
	Joint/hair supplements
Invasive interventions	Coil
	Uterine ablation
	Hysterectomy
Lifestyle changes	Exercise
	Diet / dietary changes
	Reduced alcohol intake
	Reduced caffeine intake
	Cognitive behavioural therapy / counselling
	Mindfulness/meditation
	Complementary therapies
	Work/lifestyle changes
	Menopause magnets
Temperature management	Air conditioning / fans
	Alternative clothing
	Bathing
	Alternative bedding

#### Menopause support

There was a strong association between participant role type and extent of perceived support for menopause symptoms (χ^2^(4, n = 1552) = 56.36, p <0.001): office-working staff felt more supported than shift-working colleagues at work. Furthermore, office-working staff reported being more aware of their service having a menopause policy (shift-working: 28%, n = 358; office-working: 41%, n = 152).

#### Free-text comments

**Figure 3:**
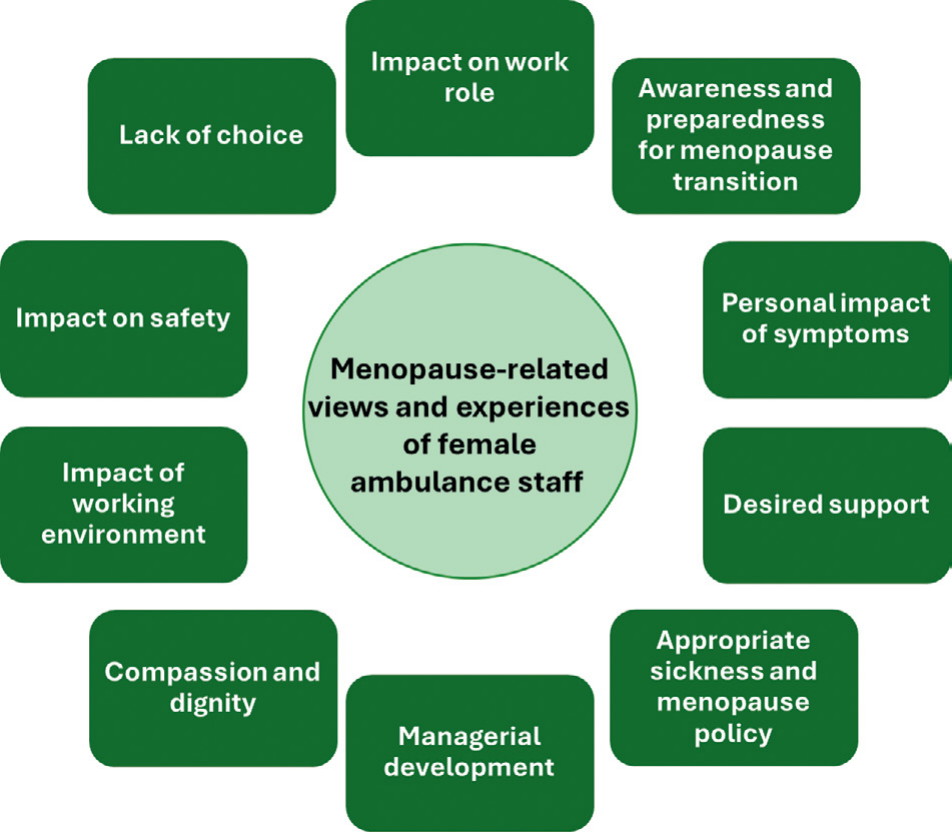
Figure 3. Main categories identified within free-text comments provided by CESSATION survey participants.

Of the 1896 participants, 664 (35%) provided free-text comments, which were analysed using a qualitative content analysis approach. Ten main categories were identified (Figure 3) and are described, with example quotations (Table 3).

**Table 3.** Example free-text quotations from CESSATION participants.

Category	Quotation	Participant
Impact on work role	*The menopause has had such a detrimental effect on my working life I decided to go from full time to part time … Regardless of being on HRT, which has alleviated some of my symptoms, I find frontline ambulance work has become far too physically demanding …*	RS816
	*The biggest problems for me have been fatigue, aching joints, hot sweats and brain fog. As an accountant I need to be awake and alert at all times, which has been really difficult with chronic brain fog.*	RO1055
Awareness and preparedness for menopause transition	*I didn’t know that I was experiencing menopause symptoms and just thought I was getting older and burnt out in my operational role.*	RS459
	*Currently the advice is there but you have to look for it, and I think I suffered longer than I needed because I didn’t know my initial symptoms were menopause related.*	RO1651
Personal impact of symptoms	*Personally struggled with memory problems and mood swings. Working with young and newly qualified staff made my symptoms worse to the point of being suicidal as they have no understanding of the issues and made me feel completely inadequate.*	RS1558
	*My menopause is that bad I regularly have iron and blood transfusions …*	RO1138
Desired support	*More published support in terms of what the service offers would be useful … that modifications to roles could be considered if applicable. An identified menopause support person(s) who could help people who need the extra support, help them to approach management with any problems, give women a confidential outlet to discuss issues and know there are other people facing the same issues as them.*	RS621
	*I am lucky I have a colleague who is similar age to me so understands what I’m going through. However, without this support I would feel totally alone at work and unable to talk about the menopause.*	RO179
Appropriate sickness and menopause policy	*I feel that my Trust has a good policy and awareness about menopause. However, I am Support staff so I would think it is easier for my line manager to manage any issues I have re. menopause symptoms than it would be if I was operational*.	RO417
	*The fear of going sick and triggering the stages of the Absence Policy, leading to having to explain at a ‘return to work’ interview with someone who does not understand, leaves me with no option other than to battle through it or make some other reason up for absence.*	RS869
Managerial development	*There should be a policy and managers should be aware that adjustments can be made. I asked my manager about not wearing uniform in the control room because I sweat in it and she laughed.*	RO450
	*Yes, there needs to be better understanding from managers regarding menopause changes and problems. There are too many male managers who don’t understand …*	RS860
Compassion and dignity	*When I told my manager that I was now in the menopause I was just sent the policy. If I bought up the subject again, I was told ‘Let’s not go there again’.*	RO810
	*A manager saying that the next thing we would want was a sign on our backs saying ‘I’m on my period’ is totally unacceptable. It’s harassment plain and simple.*	RS1748
Impact of working environment	*Menorrhagia in an emergency setting is a very significant problem for women – often in patient homes for two hours plus – where sanitary disposal is impossible.*	RS227
	*Fans at desks. Quiet rooms to sit whilst headache or migraine passes. More short breaks for drinks/toilet needs …*	RO168
Impact on safety	*… if I were still patient facing on shifts, my insomnia and concentration could make me unsafe.*	RO727
	*I have had a regular crew mate for over two decades, this really helps. I get anxiety when driving on blue lights after 30 years in the job; where he can, he drives. I get anxious about going into volatile jobs, I feel protected with a crew mate that I trust and knows me.*	RS1539
Lack of choice	*My menopause symptoms was brought on early due to breast [cancer] meds.*	RO578
	*It should not be judged as a sickness – it can’t be avoided.*	RS83

Participant RS = shift-working; RO = office-working.

### Category 1: impact on work role

Both shift- and office-working participants suggested a need for alternative employment options to help cope with symptom management, as many were concerned about menopause-related absences and subsequent disciplinary procedures or dismissal. There were some individuals considering leaving their employment or resigning due to the menopause. Alternative employment options that were suggested included reduced night shifts, shorter shifts, flexible working, alternative working duties, office-/home-based working, reduced hours contracts and early retirement.

### Category 2: awareness and preparedness for menopause transition

Menopause education and training for all workforce staff was indicated to improve awareness and understanding regarding the menopause transition. Participants identified that content should include possible menopause symptoms and their impact, as well as types of menopause, and should recognise that ‘younger’ women can experience the menopause (some menopausal participants were between 20 and 30 years). It should also support resources to include lifestyle guidance, in particular, exercise and nutrition. Manager-specific menopause training was also considered necessary.

### Category 3: personal impact of symptoms

The impact of menopause symptoms, in particular menorrhagia, flushing and sweating, could be significant, embarrassing and detrimental for both shift- and office-based participants. Despite this, participants could be reluctant or find it difficult to discuss their menopause with colleagues, including managers. Many comments suggested that the menopause is deemed a personal experience and a taboo topic, so participants could ‘suffer in silence’ rather than discuss the topic in the work environment.

### Category 4: desired support

Participants noted the current lack of workplace and expert support available to them, with most seeking support from their GP, peers, friends and family. They offered suggestions to improve menopause management and personal well-being, and suggested support should also be made available for partners and family members. Forms of support included:

- menopause support groups/networks/cafes;
- in-service dedicated menopause nurse(s);
- in-service menopause champions/guardians;
- access and referral to specialist menopause services.

### Category 5: appropriate sickness and menopause policy

This study has identified three UK ambulance services with a menopause policy and six offering menopause guidance documentation (Supplementary 1b). Survey participants identified a need for appropriate menopause policies that recognise the differing needs of both shift- and office-based staff.

Some participants questioned why the menopause could not be managed in the same way as maternity-related issues, rather than as a form of sickness. Sickness policies were often considered unsuitable for menopause-related absence, mainly due to the thresholds for triggering disciplinary stages that could lead to dismissal.

### Category 6: managerial development

Increased managerial menopause awareness and understanding, particularly by male managers, was identified by both shift- and office-based participants. Mandatory menopause transition training for managers was even suggested. The need for diversity among management teams was highlighted; a dominance of male managers was noted, and often appropriate female managers were desired for menopause support.

### Category 7: compassion and dignity

Shift- and office-based participants expressed how menopausal women and their symptoms were often seen as a joke or source of humour in the workplace. Difficulties experienced when discussing the menopause transition, particularly with male managers and younger colleagues, were highlighted. Some participants shared experiences of dismissive, derogatory and humiliating encounters with colleagues, including managers, and identified a need for appropriate disciplinary actions to ensure dignity at work, irrespective of staff level.

### Category 8: impact of working environment

Participants highlighted the importance of easy access to toilets and hygiene bins, spare uniform and the need for more and regular breaks. To facilitate temperature-related symptoms (such as hot flushes and sweating), the need for lighter, cotton-based uniforms, and alternative clothing options were also identified. In addition, access to air conditioning, fans and windows was considered important.

The physical and mental impacts of shift-working, particularly for frontline staff, were shared by participants. Many discussed how night shifts, early starts and shift patterns increased tiredness, insomnia and mental strain. They highlighted the need for work routine and stability to help with symptom management.

### Category 9: impact on safety

The penultimate category captured staff and patient safety-related concerns. Particularly frontline shift-working participants shared how sleep deprivation, exhaustion and changes in physical and mental health affected their work. They indicated that shorter shift-lengths, reduced night-working and a supportive paramedic crewmate for patient-facing staff would be beneficial.

### Category 10: lack of choice

Both shift- and office-based participants recognised the menopause transition to be a ‘natural phase’ of life that all women go through without choice, with some adopting a ‘just get on with it’ attitude for self-management. Recognising the variety and extent to which menopause symptoms can be experienced, some participants also identified the difficulties organisations face to provide appropriate menopause support for all.

## Discussion

The CESSATION study has provided an in-depth insight into the menopause transition as experienced by UK female ambulance staff. Those experiencing the peri-menopause appeared more likely to be employed in shift-working and frontline roles, whereas those in office-based roles with regular working hours appeared more likely to be in the menopause and post-menopause phases of the transition. The range of symptoms typically associated with the menopause were experienced by many and to varying extent: some women experienced mild and manageable symptoms; others had significant detrimental experiences that impacted their work and personal lives. It was observed that menopausal shift-working women reported more severe tiredness or low energy levels, hot flushes, night sweats and gastric problems, whereas office-working staff reported more severe issues with vaginal dryness, pain or itching, weight and skin changes and oral health (i.e. dry mouth and dental problems). The differences in these symptoms may provide an explanation of why shift-working staff were more likely to require leave from work when compared with their office-working colleagues. Despite an increase in public menopause-awareness campaigns, many participants reported not being prepared for their symptoms and their severity and considered the menopause transition to be a taboo experience. Some found workplace support to be lacking at peer, managerial and organisational levels, highlighting the need for appropriate menopause policies, education and support. Both shift- and office-working participants sought a wide range of self-care strategies and accessed a variety of healthcare professional support options, supporting the need for individualised approaches for menopause symptom control and support.

It is understood that certain workplace physical, psychological and organisational factors can impact experiences of menopause symptoms (Rees et al., 2021), and it has recently been recommended that the menopause be studied in relation to physical working conditions (for example, temperature and ventilation, nightshifts and physical lifting) to support women in the workplace and to understand menopause complaints and health in later life (Verdonk et al., 2022). Evidence regarding the impact of shift-working on symptom severity is limited. Cronin et al. recently reported work-related stress as a cause of increased menopause symptom severity in nursing staff. However, contrary to CESSATION findings, no correlation between shift pattern or number of hours worked and symptom severity was observed (Cronin et al., 2023); additionally, no association was reported between shift- or night-working and difficulties coping with menopausal symptoms at work in women over 50 years (D’Angelo et al., 2023). Conversely, shift- and night-working have been associated with negative effects on female reproductive health, such as menstruation, fertility and age of menopause onset (Fernandez et al., 2016; Hu et al., 2023; Khan et al., 2022; Stock et al., 2019). Given that females represent half of the ambulance workforce, with approximately one fifth of those women being over the age of 40 years (50% and 21%, respectively; see Supplementary 1a), a notable number of women are currently experiencing work-related menopause issues or will experience them in the coming years.

As workplace factors can impact the menopause experiences of women, research suggests that the menopause has a negative impact on employment for women. Increased symptom number has been reported to have an impact on work performance (Hashimoto et al., 2020), while severe symptoms, particularly vasomotor symptoms (such as hot flushes, night sweats, palpitations and mood changes), have been found to impact ability to work, job retention and level of absenteeism for menopausal women (Geukes et al., 2016; Steffan & Potočnik, 2023). CESSATION participants offered a variety of work-based suggestions to aid symptom management and promote personal well-being, work performance and retention: flexible working arrangements, fixed work patterns and alternative work roles; menopause education and training, particularly for (male) service managers; expert menopause resources and support; appropriate menopause and sickness policies for shift- and office-working staff; peer and manager support with compassion and dignity; easy access to toilet facilities and quiet areas; and availability of lighter, cotton-based uniform and cool working environments. Many of these suggestions are existing recommended interventions designed to offer menopausal women support in the workplace (Howe et al., 2023).

A key strength of the CESSATION Phase 2 survey is that the findings are consistent with those of the MIDWEST study (Prothero et al., 2021) and the CESSATION Phase 3 interviews (Brown et al., 2023), providing consistency of views and experiences of the menopause transition. As a large UK-wide study it has also enabled in-depth comparison of the menopause-related challenges faced by shift- and office-working female ambulance staff and has highlighted the need for improved national menopause support, not only to promote staff well-being and morale, but also to aid the retention of experienced and skilled workers. Key findings have been shared with the UK Association of Ambulance Chief Executives (AACE) Ambulance Leadership Forum, National Women’s Network and Uniform Working Group. Some ambulance services have now introduced new menopause policies and staff support resources. Currently, an expert stakeholder panel is being convened by AACE to consider the CESSATION findings and recommendations to determine potential interventions for evaluation in future service development and research activities.

### Limitations

Several study limitations are acknowledged. The purpose-designed survey used has yet to be formally validated (it is based on the Menopause Rating Scale (Heinemann et al., 2003), the British Menopause Society surveys (2017a, 2017b) and the MIDWEST study (Prothero et al., 2021)). In addition, as an anonymous survey, multiple entries from a single participant could not be identified unless a Phase 3 expression of interest was completed. The terms ‘female’ and ‘woman’ have been used, as almost all participants used these terms to identify their gender. Further work would be required to explore the menopause experiences of trans men and non-binary, intersex and gender-diverse people.

## Conclusion

Women working through the menopause transition in the ambulance sector experience a wide range of menopause symptoms, and whether they are shift- or office-working impacts the symptoms they experience and their need for absence from work. Appropriate workplace menopause interventions are warranted to meet the needs of this skilled workforce, promote personal well-being and aid staff retention. Further research is warranted to evaluate workplace menopause interventions and diversity in menopause.

## Author contributions

The study concept was devised by TF and LP. Both LP and TF have contributed equally to the survey design. Data analysis was undertaken by LP, TN, SB and AC. LP, with other authors providing oversight, prepared the manuscript. LP acts as the guarantor for this article.

## Conflict of interest

LP is a member of the *British Paramedic Journal* editorial board.

## Ethics

Health Research Authority approval was received (reference: 21/HRA/4564); NHS Research Ethics Committee approval was not needed due to the involvement of NHS staff only. Participants provided informed consent prior to survey completion.

## Funding

The study received funding from the College of Paramedics Small Grant Award 2020 and University of East Anglia Health and Social Care Partners.

## Patient and public involvement

The study management team included one male and two female patient and public members. Their key involvement was in the development of study materials and the interpretation of survey findings.
